# Suboptimal Serum 25-Hydroxy-Vitamin D Is Associated with a History of Recent Disease Exacerbation in Pediatric Patients with Bronchial Asthma or Asthma-Suggestive Recurrent Wheezing

**DOI:** 10.3390/ijerph17186545

**Published:** 2020-09-09

**Authors:** Teodora-Irina Adam-Bonci, Paraschiva Cherecheș-Panța, Eduard-Alexandru Bonci, Sorin Claudiu Man, Ancuța Cutaș-Benedec, Tudor Drugan, Raluca Maria Pop, Alexandru Irimie

**Affiliations:** 1 Pathophysiology, “Iuliu Hațieganu” University of Medicine and Pharmacy, 400012 Cluj-Napoca, Romania; teadaria@gmail.com; 2 3rd Pediatrics Clinic, Children’s Emergency Hospital Cluj-Napoca, 400217 Cluj-Napoca, Romania; claudiu.man@umfcluj.ro; 3 3rd Pediatrics Clinic, “Iuliu Hațieganu” University of Medicine and Pharmacy, 400217 Cluj-Napoca, Romania; 4 Oncological Surgery and Gynecologic Oncology, “Iuliu Hațieganu” University of Medicine and Pharmacy, 400015 Cluj-Napoca, Romania; airimie@umfcluj.ro; 5 Medical Informatics and Biostatistics, “Iuliu Hațieganu” University of Medicine and Pharmacy, 400349 Cluj-Napoca, Romania; cutas.ancuta@umfcluj.ro (A.C.-B.); tdrugan@umfcluj.ro (T.D.); 6 Pharmacology, Toxicology and Clinical Pharmacology, “Iuliu Hațieganu” University of Medicine and Pharmacy, 400337 Cluj-Napoca, Romania; raluca_parlog@yahoo.com

**Keywords:** vitamin D, control of asthma, asthma exacerbation prevention, child

## Abstract

Even though vitamin D is widely acknowledged as having a potential immunomodulatory role in asthma, its exact beneficial mechanisms are yet to be clarified. An optimal serum 25-hydroxy-vitamin D (25-OH-VitD) level in pediatric asthma patients might not rely solely on the effect of dose-dependent vitamin D_3_ intake, but might also be influenced by factors related to insufficient asthma control. We aimed to survey the prevalence of serum 25-OH-VitD deficiency and analyze whether suboptimal levels were associated with asthma severity factors. The current cross-sectional study enrolled 131 pediatric asthma or asthma-suggestive recurrent wheezing patients, for whom serum 25-OH-VitD, IgE, and eosinophil count were assessed. The prevalence of suboptimal serum 25-OH-VitD was 58.8%. A suboptimal vitamin D status was associated with asthma exacerbation in the previous month (*p* = 0.02). Even under seasonal oral vitamin D_3_ supplementation, patients with a positive history of asthma attack in the previous four weeks presented significantly lower serum 25-OH-VitD concentrations, compared to their peers with no disease exacerbation. In conclusion, sequential measurements of serum 25-OH-VitD might prove useful for future studies evaluating the dynamic changes in vitamin D_3_ status in regard to asthma, especially in symptomatic patients.

## 1. Introduction

Bronchial asthma is the most commonly encountered chronic condition in children worldwide [1], and has shown a growing prevalence in recent years [2]. In light of the reported increase in hospitalization for asthma exacerbation therapy [3], poor disease management seems to be a global concern; therefore, factors influencing asthma control are under intense research. Vitamin D supplementation, supposedly an accessible public health intervention, has been the subject of various types of reporting and research in relation to bronchial asthma.

The well-known immune framework of asthma describes a disbalanced reaction to allergens with the predominance of the T-helper 2 (Th2) immune reply that is intensified by the contribution of mucosal dendritic cells equipped with high-affinity immunoglobulin E receptors [4] and a defective T-helper 1 response. The paradigm also associates the flawed functions of other types of cells, such as T regulatory cells (Tregs), resulting in the lengthening of the infection course and allergic airway inflammation [4] and consequently contributing to the development of asthma.

Childhood is characterized by a fascinating process of growth and development, during which vitamin D has a crucial role in the metabolism of calcium and phosphorus, ensuring proper bone mineralization and preventing rickets [5]. However, the extra-skeletal roles have sparked interest in recent years [6], and the documentation of vitamin D receptors (VDR) in a variety of cell types [7], including immune cells, brought the immunomodulatory functions of vitamin D [8] into the spotlight. Vitamin D seems to play a part in reducing the number of infectious episodes in asthmatic patients [9]; therefore, it might have a protective role, especially in pediatric asthmatics for whom lower respiratory tract infections represent a risk factor for disease pathogenesis and may exacerbate asthma [10]. Vitamin D has been reported to reduce the exaggerated Th2 response in asthmatics [11], thus potentially influencing asthma severity.

Vitamin D is accumulated in the body, either through dietary intake in the forms of ergocalciferol (vitamin D_2_) and cholecalciferol (vitamin D_3_), or by endogenous production in the skin from 7-dehydrocholesterol under ultraviolet B action [8]. The availability of sunlight to provide sufficient exposure depends on the climate and geographical location, but is also influenced by lifestyle and the amount of time assigned for outdoor activities. For this reason, oral vitamin D_3_ supplements are recommended [12], and a considerable number of studies, including randomized controlled trials of pediatric cohorts, have reported a dose-dependent, relatively positive effect of oral vitamin D_3_ intake on the concentration of serum 25-hydroxy-vitamin D (25-OH-VitD) [13], the circulating vitamin D3 metabolite that is commonly used to investigate an individual’s vitamin D3 status [14]. In contrast, other studies have reported the opposite, namely failure to achieve the desired optimal level of serum 25-OH-VitD [15] after supplementation. However, few studies have looked into the factors that might modify the effects of vitamin D3 supplementation on the 25-OH-VitD serum level, particularly in the setting of pediatric chronic diseases.

The aim of the present study was to survey the impact of suboptimal levels of serum 25-OH-VitD in pediatric patients with asthma or recurrent wheezing suggestive of asthma in the region of Cluj County, Romania, and to analyze whether vitamin D deficiency or insufficiency is related to the factors associated with poor asthma control, particularly in patients receiving oral vitamin D_3_ supplementation.

## 2. Materials and Methods

The present report describes a cross-sectional study that was conducted between January 2019 and March 2020, that enrolled 131 pediatric patients with bronchial asthma or recurrent wheezing, registered for routine follow-up in the 3rd Pediatrics Clinic of Cluj-Napoca’s Clinical Pediatric Emergency Hospital, a tertiary unit specialized in pediatric pulmonology. The inclusion criteria were aged 0–18 years, diagnosis of bronchial asthma or recurrent wheezing suggestive of asthma according to the Global Initiative for Asthma (GINA) guidelines [16]. Exclusion criteria were decreased compliance in reporting all of the requested information, refusal to participate in the study, a diagnosis that did not meet the GINA guidelines, or any concurrent acute or chronic disease other than allergic rhinitis and atopic dermatitis. A standardized parent-addressed survey was used to record patient and family information, and physician documented files were used to collect medical data, particularly recording if the patients had received vitamin D supplementation. All parents/legal guardians consented to the participation in the study by providing an informed written consent according to the local hospital policy. This study was approved by both the ethical committee of Iuliu Hațieganu University of Medicine and Pharmacy (ethical statement number 183/30.05.2019) and of Children’s Emergency Hospital Cluj-Napoca (ethical statement number 72 SC/28.06.2019 and 9549/29.06.2020).

Patients were measured, weighed, and underwent biological work-up. Blood samples were drawn by a trained pediatric phlebotomist nurse. Complete blood count was determined using fresh venous blood collected in EDTA tubes. Samples were stored at 2–8 °C and analyzed within a maximum of four hours using an automated hematology analyzer (Mindray LabXpert 6800, Shenzhen, China). Samples for serum 25-hydroxy-vitamin D (25-OH-VitD) and total serum immunoglobulin E (IgE) testing were attained and processed in a similar manner: venous blood specimens were collected on serum separator containers, allowed to clot for thirty minutes, centrifuged within one hour, and stored at 2–8 °C. The 25-OH-VitD analysis was performed within a maximum of five hours after sampling using an automated chemiluminescence analyzer (Diasorin LIAISON XL, Sallugia, Italy). Total serum IgE was determined with commercially available ELISA kits using an automated system (Dynex Ds2, Chantilly, VA, USA). Results were evaluated and validated by a specialist physician in laboratory medicine. The cut-off value for serum 25-OH-VitD was 30 ng/mL, with values above being considered optimal, whereas both deficient (20–29.9 ng/mL) and insufficient (<20 ng/mL) values were considered suboptimal [12]. IgE was recorded as a dichotomous variable, and was considered to be elevated if it exceeded the limit for the age group (0–36 months < 10 UI/mL; 37–48 months < 25 UI/mL; 49–84 months < 50 UI/mL; 85–180 months < 100 UI/mL; 181 months and above < 150 UI/mL).

Statistical analysis was carried out using SPSS version 27.0.0 (IBM, Armonk, NY, USA). Numerical data are presented as the mean ± standard deviation or median and interquartile range (IQR). Data were evaluated for normality with the aid of the Shapiro–Wilk test, and subsequently, the Mann–Whitney U test was performed, with results reported as the median and interquartile range (IQR) with corresponding *p* values. Nominal data were reported as the frequency and percentage (*n*, %). The chi-square test was used to compare proportions, and results are presented as percentages with *p* values and, when appropriate, the relative risk was reported with the 95% confidence interval (CI).

## 3. Results

### 3.1. Characteristics of the Studied Cohort and Prevalence of Suboptimal Vitamin D Level

**Figure 1:**
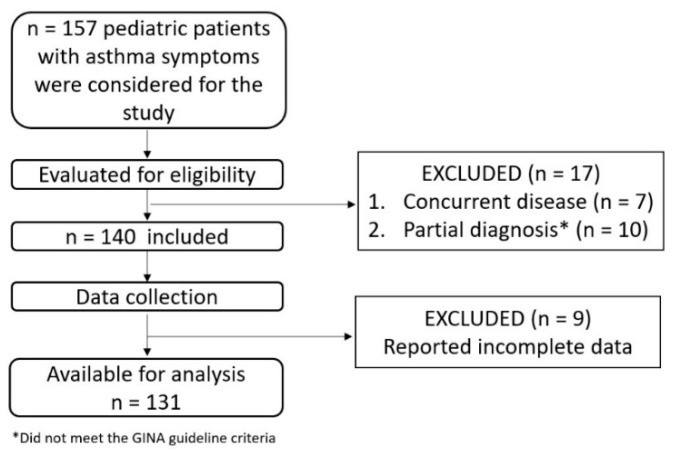
Cohort selection process.

One hundred and thirty-one patients were eligible to be included in the study, as shown in Figure 1, with the vast majority of patients (92.3%) being enrolled during the autumn, winter, and spring months. Most of the patients lived in urban areas and were born at term, as seen in Table 1. The mean age was 5.4 ± 4.03 years. The heights and weights are expressed in percentiles, which were determined using the online calculator PediTools [17]: the mean height was in the 57.48th percentile (±29.3) and the mean weight was in the 50.45th percentile (±29.16).

**Figure 2:**
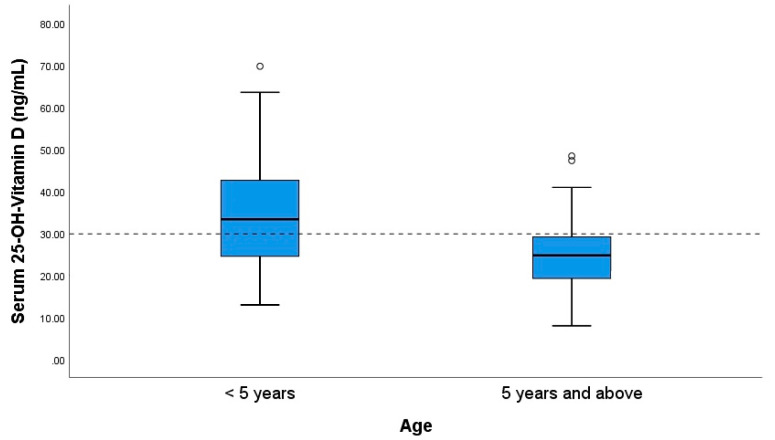
Prevalence of suboptimal serum 25-hydroxy-vitamin D (25-OH-VitD) level with significant differences between the two age groups (*p* < 0.05).

The prevalence of a suboptimal serum level of 25-OH-VitD was 58.8%. The serum level of 25-OH-VitD among children aged ≥5 years was significantly lower (U = 1210.55, *p* < 0.05) than the level of children younger than 5 years (serum 25-OH-VitD: 24.7 IQR 10.6 versus 33.3 IQR 18.17), as shown in Figure 2. For a considerable proportion of patients (80.9%, *n* = 106 of the total of 131 patients in the study), parents or caretakers declared that their child had continuously received an oral dose of 1000 I.U. cholecalciferol daily during the recommended season (from September until May).

### 3.2. Associations Analysis

Associations between the serum 25-OH-VitD status, behaviors related to the recommendations about vitamin D3 supplementation, and the status of several clinical and biological asthma severity factors are sequentially reported in Table 2. The differences in the proportions of patients with suboptimal and optimal serum 25-OH-VitD levels who responded positively to each question in Table 2 were assessed using Chi-square tests, and the results are presented as percentages with the corresponding *p* values. More patients with normal levels of serum 25-OH-VitD had received oral vitamin D3 supplements during the cold season (January–April, September–December), and more asthmatic patients with concurrent allergic rhinitis presented a suboptimal level of 25-OH-VitD, but the results had borderline statistical significance. Asthma exacerbation in the previous month was significantly associated with a suboptimal serum 25-OH-VitD level: relative risk 1.49 (95%CI 1.026–2.250), *p* = 0.02.

The analysis of the disease history in the four weeks prior to inclusion in the study, particularly for the vitamin D_3_-supplemented children, showed similar results: the group of patients that had experienced an asthma exacerbation in the last month had significantly lower (*p* = 0.047) serum 25-OH-VitD levels (median 26.6 ng/mL, IQR 19.68) compared with those in the attack-free group (median 32.2 ng/mL, IQR 16.18). However, when performing analysis according to age distribution, in the below 5 years age group, there was a statistically significant association between suboptimal 25-OH-VitD and a positive history of asthma exacerbation in the prior 4 weeks (*p* < 0.05), whereas in the group of patients aged 5 years and over, the association analysis showed only a trend towards statistical significance (*p* = 0.08).

## 4. Discussion

Prior work has explored the serum level of 25-OH-VitD [14] as a marker for vitamin D_3_ adequacy and reported decreased concentrations in pediatric asthmatics, both pre- and school-aged children. Moreover, associations between the deficiency state and various asthma characteristics have been noted [18,19,20,21]. The present study looked into vitamin D_3_ status in pediatric asthmatic and asthma-suggestive recurrent wheezing patients from the region of Cluj County in Transylvania, Romania, and analyzed whether a suboptimal serum vitamin D_3_ level was related to several factors associated with unsatisfactory control of the disease.

In our report, more than half of the patients had a suboptimal serum vitamin D_3_ level, with the observed prevalence of vitamin D deficiency/insufficiency being 58.8%, a finding similar to that outlined in other prevalence studies [18,21]. In regard to the geographical area, a previous large (*n* = 6631) cross-sectional study that evaluated the vitamin D serum level in a Romanian population of all ages (0–85 years) from all regions of the country [22] concluded that the pediatric cohort in the first decade of age presented normal vitamin D levels, with the highest mean value of serum 25-OH-VitD_3_ occurring in children under three years of age. In the same study, subjects in their second decade of life presented with serum 25-OH-VitD deficiency, but the design did not mention the associated acute or chronic illnesses at the moment of the assessment. In contrast, our current investigation explored the prevalence of suboptimal serum vitamin D_3_ levels in a cohort of 131 pediatric patients from Cluj County in Transylvania, Romania with a known diagnosis of asthma or asthma-suggestive recurrent wheezing. Consistent with the aforementioned Romanian study, our findings show that patients under the age of 5 had significantly higher levels of vitamin D_3_ compared with patients aged 5 years and above.

In the current study, 80.9% of the enrolled patients received oral vitamin D_3_ supplements during the season with inadequate environmental sun exposure, and we observed that more children with optimal serum 25-OH-VitD levels followed the supplementation regimen, but we were not able to report a statistically significant association. This may be explained by the fact that the overwhelming proportion of children (92.3%) were enrolled in our study during autumn, winter, and spring months, a period with inadequate sunlight that predisposes individuals to suboptimal serum 25-OH-VitD levels [20], making them reliant on the uptake of exogenous Vitamin D_3._ On that note, in healthy subjects, complementary vitamin D_3_ has been proven to be effective for reducing the variation in serum 25-OH-VitD across seasons by compensating for the lack of sunlight [23,24,25]. Even though the uptake of supplements produces a dose-dependent increase in 25-OH-VitD serum levels, the relationship between the two is not linear [15]. The literature emphasizes that factors such as baseline 25-OH-VitD levels [26] and age [13], even in children, may influence the degree of increase in the 25-OH-VitD serum level after vitamin D_3_ supplementation. Concerning pediatric patients with chronic diseases, our results are consistent with other reports that mention age [24] as a contributing factor to the variation in the serum vitamin D level, reasoning that, as children grow, they lack the protection of infantile rickets-preventing supplementation, while their systemic requirements increase [24]. Interestingly, bronchial asthma itself, as a comorbidity, might be a factor that influences the effect that supplemented Vitamin D_3_ has on serum 25-OH-VitD_3_ level [27]_._

We recorded several factors used to characterize the clinical course of asthma/recurrent wheezing over a twelve-month period prior to the inclusion of participants in the study, and analyzed their associations with serum 25-OH-VitD status. Oral corticoid therapy for exacerbation control and hospitalization for acute asthma attack management were not found to be associated with a suboptimal serum 25-OH-VitD status. These findings are in contrast with other studies that reported that a suboptimal serum 25-OH-VitD level is associated with increased oral corticosteroid use [28]. Further, vitamin D_3_ supplementation has been shown to lower the frequency of asthma attacks [29] and exacerbations requiring systemic corticosteroid therapy [30]. Our results might be influenced by the limited perspective associated with a singular 25-OH-VitD determination, as we did not analyze how vitamin D3 serum levels evolve in relation to disease activity and therapy interventions. 

However, considering the limited time frame used in our study, asthma exacerbations in the previous month were significantly associated with a suboptimal serum 25-OH-VitD level (Further, even in patients following an oral vitamin D_3_ supplementation regimen, those with a positive history for asthma attacks in the four weeks prior to vitamin D_3_ status quantification presented significantly lower 25-OH-VitD serum values compared with patients who were free of exacerbations. Even so, we noted that age could be a factor influencing this association.

Bronchial asthma might be associated with other allergic diseases, such as atopic dermatitis and seasonal allergic rhinitis. Atopic dermatitis (AD) is characterized by the persistent inflammation of dry skin, predisposing the individual to recurrent itching and exudative lesions, secondary to an inborn deficiency of the epithelial barrier, allowing the inoculation of pathogens and allergens. This is coupled with an imbalanced Th-2 shifted immune response that further exacerbates the lesions by predisposing the individual to wound infection. Previous studies reported that Vitamin D_3_ supposedly plays a protective, anti-infectious role, by aiding the epithelial expression of antimicrobial peptides such as cathelicidin [31], and it also plays an anti-inflammatory role by modulating the immune response, thus limiting bacterial activity and promoting the healing process. The related body of literature presents contrasting data: some studies report no significant difference in the serum 25-OH-VitD level in AD asthmatics compared with healthy children [32], whereas others have found that AD patients have lower serum 25-OH-VitD levels [33] and suggest that the presence of AD in asthmatics is a potential risk factor for suboptimal 25-OH-VitD levels [21]. In another approach in research focused on the relationship between the intensity of AD episodes and the serum concentration of 25-OH-VitD, several studies provided evidence of an inverse correlation between the two [33]. Our results revealed no association between history of AD in asthmatic or recurrent wheezing patients and a suboptimal serum level of 25-OH-VitD. A possible explanation for our finding is that we did not temporally document the occurrence of AD flares, nor their severity. Therefore, our results might be due to the fact that the patients enrolled in the study were not experiencing an AD flare at the moment of inclusion.

Another allergic disease associated with asthma, seasonal allergic rhinitis (SAR), involves the persistent inflammation of the upper airway mucosa of the nose promoted by airborne allergens that initiate and sustain an IgE-mediated response. The association between asthma and SAR is well documented [34]. Frequently, SAR might be the condition that introduces asthma [35]. Further, SAR, as an accompanying diagnosis in recurrent wheezing patients, is considered a risk factor for the initial bronchial hyperresponsiveness condition that evolves into childhood and even adult asthma [36]. An optimal serum 25-OH-vitD level seems to be an important factor that reduces the chance of SAR development [37]. In our study, we observed that more patients with asthma or recurrent wheezing with a concurrent history of SAR presented suboptimal serum 25-OH-VitD levels; however, our results were of marginally statistical significance. Similar results were reported in a meta-analysis from 2016 [38], which concluded that decreased 25-OH-VitD coexists with SAR or might be induced by SAR. One possible explanation for our finding is that patients with SAR and asthma/recurrent wheezing spend less time outdoors during the months with sufficient sunshine, a period which commonly overlaps with the seasons of increased aeroallergen concentration, thus limiting the amount of endogenous vitamin D production.

The immune signature of asthma is characterized by a predominance of the Th2 immune reply, with eosinophilia as a hallmark of the type 2 inflammation response, a well-acknowledged serological finding in allergic asthma that is also considered to be a minor criteria in the estimation of the Asthma Predictive Index [39]. Vitamin D reportedly reduces eosinophilic airway inflammation in murine lung tissue [40] and sputum from asthma patients [41]. Even though the literature reports a positive correlation between sputum and serum eosinophilia [42], several articles mention that vitamin D supplements and serum 25-OH-VitD status do not seem to significantly influence serum eosinophils [18,43], similar to the finding reported in the current study. Our results can be explained by the fact that the link between the eosinophil count and serum 25-OH-VitD was only evaluated once, at the moment that the patient was included in the study, therefore offering only a snapshot of the inflammation, whereas monitoring the dynamics of the serum levels of both might yield a more accurate reflection of the evolution of inflammation during oral vitamin D_3_ therapy [29]. Serum IgE, which is characteristic of the asthma immune response, is known to be elevated in patients with bronchial asthma, being predictive of the development of bronchial hyperreactive allergic disease in children [44], while therapy that aimed to decrease serum IgE has been shown to be beneficial for severe disease management [45]. Animal models of asthma showed that VDR-deficient asthmatic mice have significantly higher serum IgE levels, and vitamin D alongside VDR plays a role in decreasing IgE via the modulation of IL-10 secreting B cells [46,47]. Even though the literature reports an inverse correlation between serum IgE levels and the 25-OH-VitD level in child asthmatics [48], the current study did not identify any association between the total serum IgE concentration of pediatric patients with asthma and recurrent wheezing and serum 25-OH-VitD status. Our results may be explained partly by the fact that patients were not classified according to asthma phenotypes; therefore, IgE-mediated sensitization characteristic to eosinophilic asthma might not have been the main underlying pathophysiology mechanism in all patients included in the current study. In addition, total IgE was considered to be a dichotomous variable, which might prevent the phenomenon linked to the amount of increase in IgE being observed [49], considering that the literature reports that a 10 ng/mL increase in serum 25-OH-VitD produces a 25 IU decrease in serum IgE [48].

In the current study, we did not find an association between parental smoking and serum 25-OH-VitD. This finding is in contrast to other reports documenting that passive smoking is a risk factor in healthy children for suboptimal serum 25-OH-VitD levels [50]. Similar results have been found for asthmatic children, for whom exposure to tobacco smoke seems to intensify vitamin D_3_ deficiency [51]. In our study, we used parent testimony to report smoker status. However, we did not record the number of cigarettes consumed per day or the amount of indoor smoking, nor did we measure markers of tobacco exposure, such as urinary cotinine [52], in children to objectively assess the amount of second-hand smoke exposure in our cohort. Furthermore, parents of asthmatic children in particular are exhaustively informed about the negative effects of their habit on their child’s disease; therefore, parents who smoke might avoid this behavior in the presence of their child. Thus, a possible justification for our finding might be related to parental attitude towards smoking, but our data recording does not allow us to report this.

This study has several limitations. First of all, it was a cross-sectional, observational study, including patients that presented to the clinic for asthma or recurrent wheezing evaluation and diagnosis; therefore, the reported results reflect a limited image of the real phenomenon and lack cause and effect assumptions. The design did not include a control group without asthma, but literature reports of serum vitamin D_3_ were cited for comparison. Furthermore, data collection involved parent/legal guardian questionnaires; therefore, a recall bias should be assumed, even if information was supplemented from standardized physician-covered medical records. Secondly, the cohort included a limited number of patients that were very heterogenous regarding age [13] and moment of asthma onset and disease development [53], factors that might influence the observed 25-OH-VitD serum levels. Thirdly, asthma and disease exacerbation characteristics were observed over a twelve-month period, but there was only one measurement of the 25-OH-VitD level, at the moment of inclusion in the study. Using a single measurement of 25-OH-VitD to investigate an association may have failed to comprehensively determine how the serum vitamin D_3_ status varied in regard to the considered asthma characteristics.

## 5. Conclusions

The prevalence of a suboptimal serum level of 25-OH-VitD in the studied cohort was 58.8%, similar to that reported in the literature. Asthmatic and asthma-suggestive recurrent wheezing patients with a positive history of disease exacerbation in the previous month were more likely to have suboptimal 25-OH-VitD levels.

This finding suggests that further studies taking into account sequential determination of serum 25-OH-VitD might be useful for assessing the dynamics of the vitamin D3 status in symptomatic asthmatic and asthma-suggestive wheezing patients. This approach could offer insight into identifying the factors interfering with the ability of vitamin D3 therapy to achieve the desired optimal serum 25-OH-VitD level in these patients, as a step towards the development of tailored disease management.

## Figures and Tables

**Table 1:** Social and demographic features of the studied cohort.

	Age		Gender		Residence		Premature Birth	
	<5 Years	≥5 Years	Female	Male	Urban	Rural	Yes	No
n	70	61	60	71	99	32	24	107
%	53.4	46.6	45.8	54.2	75.6	24.4	18.3	81.7

**Table 2:** Behavioral, clinical, biological, and therapy characteristics of the asthma and asthma-suggestive recurrent wheezing patients, reported in relation to Vitamin D status, assessed through the serum 25-hydroxy-vitamin D_3_ level.

Question	Suboptimal Serum 25-OH-VitD n = 77	Optimal Serum 25-OH-VitD n = 54	*p* Value
Vitamin D_3_ supplementation in infancy	83.1% (64/77)	90.7% (49/54)	0.21
Vitamin D3 supplementation in the cold season	75.3% (58/77)	88.8% (48/54)	0.052
Parental smoking	32.4% (25/77)	37% (20/54)	0.58
Personal history of atopic dermatitis	54.5% (42/77)	61.1% (33/54)	0.45
Personal history of seasonal allergic rhinitis	38.5% (22/77)	14.8% (8/54)	0.06
Elevated-for-age group serum IgE ^1^	44.1% (34/77)	57.4% (31/54)	0.13
Eosinophil count >4% of total white blood count ^1^	31.1% (24/77)	27.7% (15/54)	0.23
Hospitalization for asthma attack ^2^	22.0% (17/77)	11.1% (6/54)	0.1
Oral corticosteroids for exacerbation therapy ^2^	37.6% (29/77)	27.7% (15/54)	0.23
Recent asthma exacerbation ^3^	61.0% (47/77)	40.7% (22/54)	0.02

^1^ Assessed at the time of inclusion in the study; ^2^ Occurred at least once in the last twelve months prior to enrollment in the study; ^3^ Occurred at least once at any time in the four weeks prior to enrollment in the study.

## References

[1] Serebrisky D., Wiznia A. (2019). Pediatric Asthma: A Global Epidemic. Ann. Glob. Health.

[2] Mallol J., Crane J., von Mutius E., Odhiambo J., Keil U., Stewart A. (2013). The International Study of Asthma and Allergies in Childhood (ISAAC) Phase Three: A Global Synthesis. Allergol. Immunopathol. (Madr).

[3] Masoli M., Fabian D., Holt S., Beasley R. (2004). The Global Burden of Asthma: Executive Summary of the GINA Dissemination Committee Report. Allergy Eur. J. Allergy Clin. Immunol..

[4] Raedler D., Schaub B. (2014). Immune Mechanisms and Development of Childhood Asthma. Lancet Respir. Med..

[5] Christakos S., Dhawan P., Verstuyf A., Verlinden L., Carmeliet G. (2015). Vitamin D: Metabolism, Molecular Mechanism of Action, and Pleiotropic Effects. Physiol. Rev..

[6] Saggese G., Vierucci F., Boot A.M., Czech-Kowalska J., Weber G., Camargo C.A., Mallet E., Fanos M., Shaw N.J., Holick M.F. (2015). Vitamin D in Childhood and Adolescence: An Expert Position Statement. Eur. J. Pediatr..

[7] Wang Y., Zhu J., DeLuca H.F. (2012). Where Is the Vitamin D Receptor?. Arch. Biochem. Biophys..

[8] Gil Á., Plaza-Diaz J., Mesa M.D. (2018). Vitamin D: Classic and Novel Actions. Ann. Nutr. Metab..

[9] Ramos-Martínez E., López-Vancell M.R., de Córdova-Aguirre J.C.F., Rojas-Serrano J., Chavarría A., Velasco-Medina A., Velázquez-Sámano G. (2018). Reduction of Respiratory Infections in Asthma Patients Supplemented with Vitamin D Is Related to Increased Serum IL-10 and IFNγ Levels and Cathelicidin Expression. Cytokine.

[10] Oddy W.H., de Klerk N.H., Sly P.D., Holt P.G. (2002). The Effects of Respiratory Infections, Atopy, and Breastfeeding on Childhood Asthma. Eur. Respir. J..

[11] Pfeffer P.E., Chen Y.H., Woszczek G., Matthews N.C., Chevretton E., Gupta A., Saglani S., Bush A., Corrigan C., Cousins D.J. (2015). Vitamin D Enhances Production of Soluble ST2, Inhibiting the Action of IL-33. J. Allergy Clin. Immunol..

[12] Saggese G., Vierucci F., Prodam F., Cardinale F., Cetin I., Chiappini E., De Angelis G.L., Massari M., Del Giudice E.M., Del Giudice M.M. (2018). Vitamin D in Pediatric Age: Consensus of the Italian Pediatric Society and the Italian Society of Preventive and Social Pediatrics, Jointly with the Italian Federation of Pediatricians. Ital. J. Pediatr..

[13] Mo M., Wang S., Chen Z., Muyiduli X., Wang S., Shen Y., Shao B., Li M., Chen D., Chen Z. (2019). A Systematic Review and Meta-Analysis of the Response of Serum 25-Hydroxyvitamin D Concentration to Vitamin D Supplementation from RCTs from around the Globe. Eur. J. Clin. Nutr..

[14] Hollis B.W., Wagner C.L., Drezner M.K., Binkley N.C. (2007). Circulating Vitamin D3 and 25-Hydroxyvitamin D in Humans: An Important Tool to Define Adequate Nutritional Vitamin D Status. J. Steroid Biochem. Mol. Biol..

[15] Mazahery H., von Hurst P.R. (2015). Factors Affecting 25-Hydroxyvitamin D Concentration in Response to Vitamin D Supplementation. Nutrients.

[16] Global Initiative for Asthma (2020). Global Stategy for Asthma Management and Prevention.

[17] Care C., Improvement Q. (2020). PediTools Electronic Growth Chart Calculators: Applications in Clinical Care, Research, and Quality Improvement. J. Med. Internet Res..

[18] Licari A., Marseglia G.L., Ciprandi G. (2019). Vitamin D3 in Children with Allergic Asthma in Clinical Practice. Pediatr. Pulmonol..

[19] Bose S., Diette G.B., Woo H., Koehler K., Romero K., Rule A.M., Detrick B., Brigham E., McCormack M.C., Hansel N.N. (2019). Vitamin D Status Modifies the Response to Indoor Particulate Matter in Obese Urban Children with Asthma. J. Allergy Clin. Immunol. Pract..

[20] Özdoğan Ş. (2019). Seasonal, Sex Variations in Vitamin d Levels and Their Association with Pulmonary Function in Children with Asthma. Turk. J. Med. Sci..

[21] Peçanha M.B., Freitas R.B., Moreira T.R., Silva L.S., de Oliveira L.L., Cardoso S.A. (2019). Prevalence of Vitamin D Deficiency and Its Relationship with Factors Associated with Recurrent Wheezing. J. Bras. Pneumol..

[22] Chirita-Emandi A., Socolov D., Haivas C., Calapiş A., Gheorghiu C., Puiu M. (2015). Vitamin D Status: A Different Story in the Very Young versus the Very Old Romanian Patients. PLoS ONE.

[23] Pittaway J.K., Ahuja K.D.K., Beckett J.M., Bird M.L., Robertson I.K., Ball M.J. (2013). Make Vitamin D While the Sun Shines, Take Supplements When It Doesn′t: A Longitudinal, Observational Study of Older Adults in Tasmania, Australia. PLoS ONE.

[24] Holmlund-Suila E., Koskivirta P., Metso T., Andersson S., Mäkitie O., Viljakainen H.T. (2013). Vitamin D Deficiency in Children with a Chronic Illness-Seasonal and Age-Related Variations in Serum 25-Hydroxy Vitamin D Concentrations. PLoS ONE.

[25] Mavroeidi A., Aucott L., Black A.J., Fraser W.D., Reid D.M., Macdonald H.M. (2013). Seasonal Variation in 25(OH)D at Aberdeen (57°N) and Bone Health Indicators- Could Holidays in the Sun and Cod Liver Oil Supplements Alleviate Deficiency?. PLoS ONE.

[26] Kaykhaei M.A., Khodadoost M., Dashipour A.R., Haidari Z., Karimkoshteh A., Sandoughi M. (2019). Baseline Levels Determine Magnitude of Increment in 25 Hydroxy Vitamin D Following Vitamin D3 Prescription in Healthy Subjects. Endocrine.

[27] Wu Z., Camargo C.A., Reid I.R., Beros A., Sluyter J.D., Waayer D., Lawes C.M.M., Toop L., Khaw K., Scragg R. (2020). What Factors Modify the Effect of Monthly Bolus Dose Vitamin D Supplementation on 25-Hydroxyvitamin D Concentrations?. J. Steroid Biochem. Mol. Biol..

[28] Searing D.A., Zhang Y., Murphy J.R., Hauk P.J., Goleva E., Leung D.Y.M. (2010). Decreased Serum Vitamin D Levels in Children with Asthma Are Associated with Increased Corticosteroid Use. J. Allergy Clin. Immunol..

[29] Solidoro P., Bellocchia M., Aredano I., Mattei A., Pivetta E., Patrucco F., Boita M., de Blasio F., Brussino L., Rolla G. (2017). Asthmatic Patients with Vitamin D Deficiency Have Decreased Exacerbations after Vitamin Replacement. Nutrients.

[30] Martineau A.R., Jolliffe D.A., Greenberg L., Hooper R.L., Griffiths C.J., Camargo C.A., Kerley C.P., Jensen M.E., Mauger D., Stelmach I. (2017). Vitamin D Supplementation to Prevent Asthma Exacerbations: A Systematic Review and Meta-Analysis of Individual Participant Data. Lancet Respir. Med..

[31] Hata M.D.T.R., Kotol B.S.P., Jackson M.D.M., Nguyen B.S.M., Paik M.D.A., Don Udall M.D., Kanada B.S.K., Yamasaki M.D.K., Alexandrescu M.D., Richard L. (2008). Administration of Oral Vitamin D Induces Cathelicidin Production in Atopic Individuals. J. Allergy Clin. Immunol..

[32] Reinehr T., Langrock C., Hamelmann E., Lücke T., Koerner-Rettberg C., Holtmann M., Legenbauer T., Gest S., Frank M., Schmidt B. (2018). 25-Hydroxvitamin D Concentrations Are Not Lower in Children with Bronchial Asthma, Atopic Dermatitis, Obesity, or Attention-Deficient/Hyperactivity Disorder than in Healthy Children. Nutr. Res..

[33] Wang S.S., Hon K.L., Kong A.P., Pong H.N.S., Wong G.W.H., Leung T.F.K. (2014). Vitamin D Deficiency Is Associated with Diagnosis and Severity of Childhood Atopic Dermatitis. Pediatr. Allergy Immunol..

[34] Bousquet J., Schünemann H.J., Samolinski B., Demoly P., Baena-Cagnani C.E., Bachert C., Bonini S., Boulet L.P., Bousquet P.J., Brozek J.L. (2012). Allergic Rhinitis and Its Impact on Asthma (ARIA): Achievements in 10 Years and Future Needs. J. Allergy Clin. Immunol..

[35] Di Cara G., Carelli A., Latini A., Panfili E., Bizzarri I., Ciprandi G., Buttafava S., Frati F., Verrotti A. (2015). Severity of Allergic Rhinitis and Asthma Development in Children. World Allergy Organ. J..

[36] Sears M.R. (2015). Predicting Asthma Outcomes. J. Allergy Clin. Immunol..

[37] Aryan Z., Rezaei N., Camargo C.A. (2017). Vitamin D Status, Aeroallergen Sensitization, and Allergic Rhinitis: A Systematic Review and Meta-Analysis. Int. Rev. Immunol..

[38] Kim Y.H., Kim K.W., Kim M.J., Sol I.S., Yoon S.H., Ahn H.S., Kim H.J., Sohn M.H., Kim K.E. (2016). Vitamin D Levels in Allergic Rhinitis: A Systematic Review and Meta-Analysis. Pediatr. Allergy Immunol..

[39] Castro-Rodríguez J.A., Holberg C.J., Wright A.L., Martinez F.D. (2000). A Clinical Index to Define Risk of Asthma in Young Children with Recurrent Wheezing. Am. J. Respir. Crit. Care Med..

[40] Vasiliou J.E., Lui S., Walker S.A., Chohan V., Xystrakis E., Bush A., Hawrylowicz C.M., Saglani S., Lloyd C.M. (2014). Vitamin D Deficiency Induces Th2 Skewing and Eosinophilia in Neonatal Allergic Airways Disease. Allergy Eur. J. Allergy Clin. Immunol..

[41] De Groot J.C., Van Roon E.N.H., Storm H., Veeger N.J.G.M., Zwinderman A.H., Hiemstra P.S., Bel E.H.D., Brinke A.T. (2015). Vitamin D Reduces Eosinophilic Airway Inflammation in Nonatopic Asthma. J. Allergy Clin. Immunol..

[42] Jolliffe D.A., Kilpin K., MacLaughlin B.D., Greiller C.L., Hooper R.L., Barnes N.C., Timms P.M., Rajakulasingam R.K., Bhowmik A., Choudhury A.B. (2018). Prevalence, Determinants and Clinical Correlates of Vitamin D Deficiency in Adults with Inhaled Corticosteroid-Treated Asthma in London, UK. J. Steroid Biochem. Mol. Biol..

[43] Doǧru M., Seren L.P. (2017). Serum 25-Hydroxyvitamin D Levels in Children with Recurrent Wheezing and Relation to the Phenotypes and Frequency of Wheezing. Eur. Ann. Allergy Clin. Immunol..

[44] Lama M., Chatterjee M., Chaudhuri T.K. (2013). Total Serum Immunoglobulin e in Children with Asthma. Indian J. Clin. Biochem..

[45] Abraham I., Alhossan A., Lee C.S., Kutbi H., MacDonald K. (2016). “Real-Life” Effectiveness Studies of Omalizumab in Adult Patients with Severe Allergic Asthma: Systematic Review. Allergy Eur. J. Allergy Clin. Immunol..

[46] James J., Weaver V., Cantorna M.T. (2017). Control of Circulating IgE by the Vitamin D Receptor In Vivo Involves B Cell Intrinsic and Extrinsic Mechanisms. J. Immunol..

[47] Sandhu M.S., Casale T.B. (2010). The Role of Vitamin D in Asthma. Ann. Allergy, Asthma Immunol..

[48] Brehm J.M., Celedón J.C., Soto-Quiros M.E., Avila L., Hunninghake G.M., Forno E., Laskey D., Sylvia J.S., Hollis B.W., Weiss S.T. (2009). Serum Vitamin D Levels and Markers of Severity of Childhood Asthma in Costa Rica. Am. J. Respir. Crit. Care Med..

[49] Simpson A., Soderstrom L., Ahlstedt S., Murray C.S., Woodcock A., Custovic A. (2005). IgE Antibody Quantification and the Probability of Wheeze in Preschool Children. J. Allergy Clin. Immunol..

[50] Nwosu B.U., Kum-Nji P. (2018). Tobacco Smoke Exposure Is an Independent Predictor of Vitamin D Deficiency in US Children. PLoS ONE.

[51] Chinellato I., Piazza M., Sandri M., Paiola G., Tezza G., Boner A.L. (2018). Correlation between Vitamin D Serum Levels and Passive Smoking Exposure in Children with Asthma. Allergy Asthma Proc..

[52] Protano C., Andreoli R., Mutti A., Manigrasso M., Avino P., Vitali M. (2018). Reference Intervals for Urinary Cotinine Levels and the Influence of Sampling Time and Other Predictors on Its Excretion among Italian Schoolchildren. Int. J. Environ. Res. Public Health.

[53] McKenna S.H., Fenton T.R., Noseworthy M., Anselmo M. (2016). Adequate Vitamin D Intake but Low Serum Levels in Pediatric Asthma Patients: A Pilot Study, Alberta Children’s Hospital. Can. Respir. J..

